# *Calleida desenderi*, new species from Ecuador (Coleoptera, Carabidae, Lebiinae)

**DOI:** 10.3897/zookeys.100.1522

**Published:** 2011-05-20

**Authors:** Achille Casale

**Affiliations:** 1 Università di Sassari, Dipartimento di Zoologia e Genetica Evoluzionistica, Via Muroni 25, 07100 Sassari (Italy)

**Keywords:** Coleoptera, Carabidae, *Calleida desenderi*, new species, Ecuador

## Abstract

*Calleida desenderi* Casale, **sp. n.**, is described from Ecuador, Napo Province, surroundings of San Rafael. The new taxon is mostly characterized by the head and appendages rufous, the disc of elytra with marked metallic green reflection, the median lobe of aedeagus ring-like, and the endophallus with a long, twisted flagellum. A key for identification of the closer Neotropical species described so far is also provided.

## Introduction

As I recently noted (Casale 2008), Ecuador, in spite of its small surface area (283,561 square kms), is a South American country that includes a surprising variety of environments. Thanks to its geographical position crossed by the Equator, the occurrence of some of the highest peaks of Southern America, and the presence of tropical rain and cloud forests on both the Pacific and Amazon sides of the Andes, Ecuador is currently cited as one of the main hot-spots of biodiversity and endemism in the world, but also as one of the areas more threatened by deforestation. However, its mainland insect fauna, with some exceptions (see Moret 2005), is little known at present. Amongst carabid beetles, the genus *Calleida* Dejean 1825 (in the widest sense of Chaudoir 1972), including nice, arboreal species mostly tied to wet forests, can be cited as a good example of scarce knowledge.

On the contrary, the Galapagos islands and their biodiversity offer a fantastic place for any student familiar with evolutionary biology, and have been investigated for many decades. Several contributions from these investigations include beetles (for a synopsis see Peck 2006).

Konjev Desender and Jean Pierre Maelfait contributed greatly to the knowledge of the arthropod fauna of the Galapagos Islands: thanks to many travels, they had the opportunity to produce a series of highly interesting scientific contributions dedicated to this archipelago (see Lövei 2011 for a list of Konjev’s publications).

Thanks to Konjev, I had the opportunity to describe the only *Calleida* species known so far in Galapagos (*Calleida migratoria* Casale in Desender et al. 2002), a species introduced from Peru and now widely spread on several islands. Therefore, this is for me an honour – but also a great sadness - to dedicate to him this paper, and a very fine and interesting *Calleida* species from Ecuador, in memory of pleasant days spent with him in congresses, in the field, and at his Institute in Brussels.

## Material and methods

The following data come from many specimens of Neotropical *Calleida* species examined so far (including type series), most of them received from different museums, institutes and colleagues. The type material of the new species here described has been offered to me for study by my good friend Giovanni Onore, former professor of Entomology at the Pontificia Universidad Catolica del Ecuador in Quito.

Male genitalia were dissected, dehydrated in ethanol, cleared in cold KOH, examined and illustrated, using standard techniques before their definitive inclusion on microscope slides. Line drawings were made using a camera lucida attached to stereomicroscopes Wild M-3 and Wild M-5, and a microscope Leitz Orthoplan. The photograph of habitus was obtained using a digital camera Canon G6 attached to stereomicroscope Zeiss Stemi 2000.

### Acronyms:

TL	body Total Length, from the anterior margin of clypeus to the apex of elytra, measured along the suture.

L	overall Length, from apex of mandibles to apex of elytra, measured along the suture.

PL/PW	ratio Length of Pronotum, as linear distance from the anterior to the basal margin, measured along the midline/maximum Width of Pronotum, as greatest transverse distance.

EL/EW	ratio Length of Elytra, as linear distance from the basal ridge to the apex, measured along the suture/maximum Width of Elytra.

### Collections:

QCAZ	Zoology Museum, Departamento de Biologia, Pontificia Universidad Catolica del Ecuador, Quito (Ecuador)

CCa	Casale collection, University of Sassari (Italy)

## Taxonomic treatment and morphological terms

In this contribution, the genus *Calleida* is treated in the narrow sense, i.e. as a unit including only American species, and excluding African and Asiatic taxa (*Callidiola* Jeannel, 1949, *Stenocallida* Jeannel, 1949, of authors), currently treated at subgeneric rank of *Calleida* (see, among others: Lorenz 2005). In spite of this choice, the limits of these generic groups are not yet defined.

The median lobe of aedeagus is a synonym of phallus of some authors. Endophallus is synonym of inner sac of authors.

### Calleida desenderi

Casale sp. n.

urn:lsid:zoobank.org:act:D9A8E5CC-04AB-4F26-AB9E-BAB0216BD3F8

http://species-id.net/wiki/Calleida_desenderi

#### Diagnosis.

With the character states of the Neotropical *Calleida* species (see Erwin 2004), but markedly characterised by the peculiar combination of the following morphological features: medium sized (L: 9.0–9.5 mm; TL: 8.5–9.0 mm); body and appendages rufous, contrasting in colour with the translucent, metallic green disc of elytra; pronotum slightly transverse, with lateral margins slightly sinuate in the basal fourth; elytra moderately elongate, depressed, with marked pre-apical callosity and apical margin not beaded, bent and prominent at the sutural angle. Abdominal sternum VII with two setae on each side in males, three setae in females.

Male genitalia as in Figs 3–5: median lobe of aedeagus ring-like, depressed at sides; apex short; endophallus with a long, twisted flagellum.

**Figure 1.**
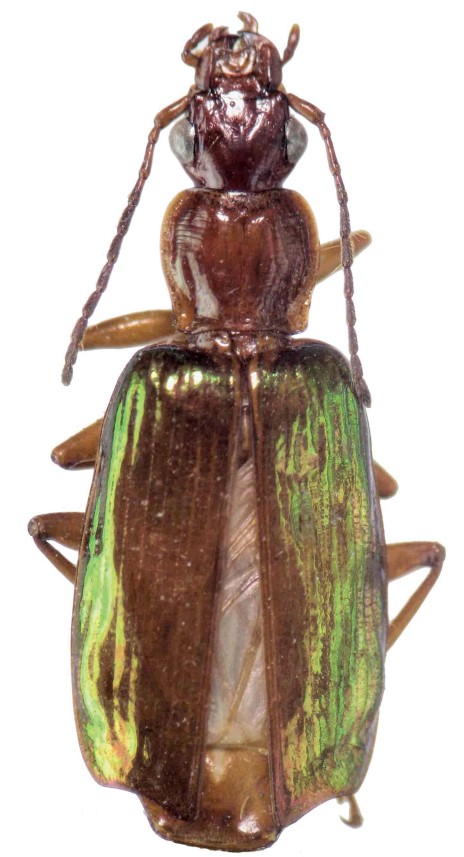
*Calleida desenderi* sp. n., female paratype, habitus, dorsal aspect.

Close to *Calleida scyntillans* Bates, 1883 and *Calleida schumacheri* Steinheil, 1875, *Calleida desenderi* sp. n. is distinguishable from the closest Neotropical species for the features stressed below (see Key, in Relationships).

#### Type locality.

Ecuador, Napo: San Rafael, 1400 m.

#### Type material.

Holotype ♂: Ecuador, Napo: Reventador, San Rafael 1400 m, 10 Jan 1998 F. Maza, at light trap (QCAZ); paratype ♀: Ecuador, Sucumbios, Cascada San Rafael, 1150 m, 77°33'30"W, 00°2'44"S, 30 Apr 2004, D. Cisneros (CCa).

Note: the male holotype presents some malformations (elytral intervals wrinkled, metatibiae asymmetrically curved). For this very reason, the female paratype is illustrated in Fig. 1.

#### Etymology and dedication.

It is a great honor for me to dedicate this new Ecuadorian species to the memory of Konjev Desender, the Belgian carabidologist who contributed greatly to the knowledge of carabids of the Galapagos Islands, the famous archipelago belonging to the Republic of Ecuador

#### Description.

General features as in Fig. 1. Medium sized: L: 9.0 mm (male holotype) – 9.5 mm (female paratype); TL: mm 8.5 mm (male holotype) – 9.0 mm (female paratype).

*Colour:* Head, base of antennae, prothorax, pterothorax, abdomen, basal and lateral margins of elytra, and legs, concolorous pale rufous; apex of mandibles, apical half of antennomere 4, and antennomeres 5–11 markedly infuscated; disc of elytra translucent, light metallic golden green (but reddish at oblique light), with cupreous-purple reflections at apex and on the sutural interval.

*Lustre and microsculpure:* Head and pronotum shiny, with highly effaced microsculpture; elytra shiny, translucent, with fine, hardly visible reticulate sculpture and marked metallic lustre.

*Head:* wide, with moderate neck constriction; genae short, moderately swollen and regularly curved to the neck constriction, not contiguous with the posterior margin of eyes; frontal furrows sparsely punctuate; eyes very large and prominent; two supraorbital setae on each side.

*Prothorax:* subquadrate, slightly wider than long (ratio PL/PW: 0.9), with lateral sides shortly sinuate in the basal fourth. Lateral reflection moderate, more evident basally; lateral furrows wide, depressed, each with a series of deep punctures. Disc moderately depressed, with marked transversal wrinkles. Anterior angles rounded, not prominent; basal angles obtuse. Basal margin markedly oblique at the extreme lateral sides. One paramedial seta and one basolateral seta on each side present.

*Elytra:* moderately elongate (ratio EL/EW: 1.7), slightly widened at the apical third; disc depressed, with evident concavity at the middle on each elytron; striae superficial, shallowly punctuate; intervals flat. Post-humeral sinuation shallow, pre-apical outer callosity evident on intervals 7–8. Apical margin obliquely bent, markedly prominent at the sutural angle, not beaded. Interval 3 with two small discal and one apical setiferous pores; umbilicate series of 13 pores along stria 8.

*Hind wings:* fully developed.

*Legs:* femora robust, tibiae elongate, tarsomeres of slender form; only metatarsomere 1 grooved dorsally; metatarsomere 4 deeply bilobed, its lobes short, widened and truncate at apex. Tarsal claws denticulate, each with six long teeth on the inner side.

**Figure 2-5.**
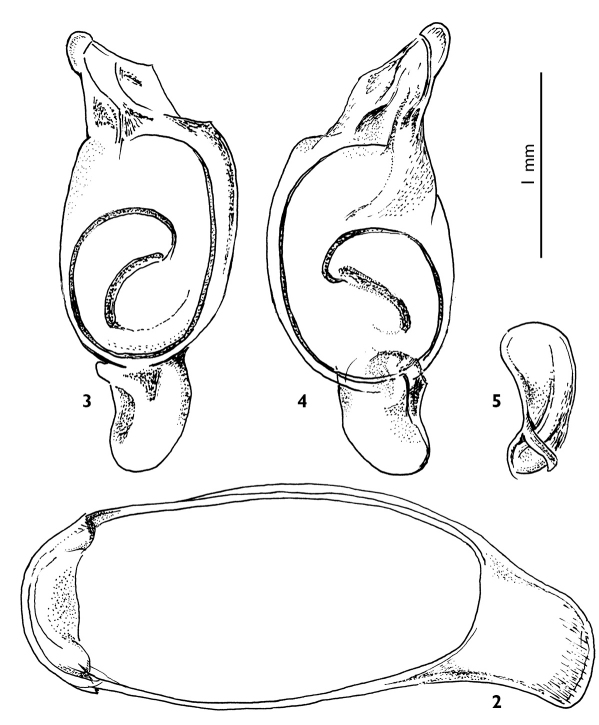
*Calleida desenderi* sp. n., male holotype **2** abdominal segment IX, ring sclerite **3** male genitalia, median lobe of aedeagus and inner sac, dorsal-right aspect **4** male genitalia, median lobe of aedeagus and inner sac, lateral left aspect **5** male genitalia, left paramere.

*Abdominal sterna:* sternum VII with two setae on each side in males, three setae in females; male abdominal segment IX as in Fig. 2.

*Male genitalia:* median lobe of aedeagus (Figs 3–4) ring-like, depressed at sides; apex short, distally rounded; endophallus with a long, twisted flagellum. Left paramere as in Fig. 5.

*Female genitalia:* not examined, owing to the fact that the abdominal sterna, in the only female specimen known so far, were not fully sclerotized.

#### Geographical distribution and ecology.

*Calleida desenderi* sp. n.is known so far from Ecuador (Napo Province), surroundings of San Rafael, on the Amazon side of the Andes. The two specimens of the type series were obtained in January and April, in two different years, in secondary humid premontane forests at 1150–1400 m.

#### Relationships.

The most interesting and curious fact is that *Calleida desenderi* sp. n. is very similar in external features to the sympatric species *Calleida vignai* Casale, 2008, this also known so far from only two individuals sampled in two different years. This datum confirms the well known occurrence, in arboreal canopies of tropical forests, of apparently rare sibling species belonging to different species groups, markedly isolated by pre-zygotic barriers thanks to different phenologies, habitat choices, and by completely different morphological features in genitalia.

In fact, *Calleida vignai*, for the character state of male genitalia (median lobe of aedeagus elongate and slender, endophallus with copulatory lamella composed by two pieces connected at the base), belongs to a group of taxa that I indicated as *lindigii* species group (Casale 1988, 2008).

On the contrary, *Calleida desenderi* sp. n. belongs to another group of Neotropical species, that here I will indicate as *cupreocincta* species group. Diagnostic features of this group are: body and appendages rufous, elytra in part or fully metallic green; elytra moderately elongate, depressed, with apical margin not beaded. Abdominal sternum VII with two setae on each side in males, three setae in females. Male genitalia: median lobe of aedeagus ring-like, depressed at sides; endophallus with a long, twisted flagellum.

The group includes some not yet described species from Central and Southern America. The species described so far can be distinguished by the following key:

**Table:** 

1	Genae markedly swollen, abruptly constricted to the neck. Elytral disc mostly dark rufous, with metallic green reflection only at base and on outer intervals 7–9. Elytral pre-apical callosity slightly distinct. Metatarsomeres 1–3 deeply grooved dorsally. Range: Brazil, Atlantic coast (Pernambuco, Rio de Janeiro)	*Calleida cupreocincta* Chaudoir, 1848
–	Genae swollen but regularly curved, not abruptly constricted to the neck. Elytral disc with metallic reflection extended to all intervals. Elytral pre-apical callosity markedly distinct on intervals 7–8. Metatarsomere 3 not grooved dorsally	2
2	Pronotum elongate, constricted in front. Elytral disc with marked metallic green reflection, but with distinct reddish patch on the inner intervals, more evident at oblique light. Metatarsomeres 1–2 superficially grooved dorsally. Range: Central America (Panama)	*Calleida scyntillans* Bates, 1883
–	Pronotum subquadrate or slightly transverse. Elytral disc fully metallic green or golden green. Only metatarsomere 1 grooved dorsally	3
3	Apical half of antennomere 1, and following antennomeres, markedly infuscated. Apical margin and sutural interval of elytra with metallic cupreous-purple reflection. Range: Ecuador (Napo)	*Calleida desenderi* Casale, sp. n.
–	Antennae fully rufous. Sutural interval of elytra bright metallic green; apical margin of elytra yellow reddish, as the base and lateral margins. Range: Colombia	*Calleida schumacheri* Steinheil, 1875

## Supplementary Material

XML Treatment for Calleida desenderi
